# Unbiased identification of novel subclinical imaging biomarkers using unsupervised deep learning

**DOI:** 10.1038/s41598-020-69814-1

**Published:** 2020-07-31

**Authors:** Sebastian M. Waldstein, Philipp Seeböck, René Donner, Amir Sadeghipour, Hrvoje Bogunović, Aaron Osborne, Ursula Schmidt-Erfurth

**Affiliations:** 1 Christian Doppler Laboratory for Ophthalmic Image Analysis, Vienna Reading Center, Department of Ophthalmology and Optometry, Medical University Vienna, Waehringer Guertel 18-20, 1090 Vienna, Austria; 2 Genentech, Inc, 1 DNA Way, South San Francisco, CA USA

**Keywords:** Macular degeneration, Scientific data

## Abstract

Artificial intelligence has recently made a disruptive impact in medical imaging by successfully automatizing expert-level diagnostic tasks. However, replicating human-made decisions may inherently be biased by the fallible and dogmatic nature of human experts, in addition to requiring prohibitive amounts of training data. In this paper, we introduce an unsupervised deep learning architecture particularly designed for OCT representations for unbiased, purely data-driven biomarker discovery. We developed artificial intelligence technology that provides biomarker candidates without any restricting input or domain knowledge beyond raw images. Analyzing 54,900 retinal optical coherence tomography (OCT) volume scans of 1094 patients with age-related macular degeneration, we generated a vocabulary of 20 local and global markers capturing characteristic retinal patterns. The resulting markers were validated by linking them with clinical outcomes (visual acuity, lesion activity and retinal morphology) using correlation and machine learning regression. The newly identified features correlated well with specific biomarkers traditionally used in clinical practice (r up to 0.73), and outperformed them in correlating with visual acuity ($\hbox {R}^{2} = 0.46$ compared to $\hbox {R}^{2} = 0.29$ for conventional markers), despite representing an enormous compression of OCT imaging data (67 million voxels to 20 features). In addition, our method also discovered hitherto unknown, clinically relevant biomarker candidates. The presented deep learning approach identified known as well as novel medical imaging biomarkers without any prior domain knowledge. Similar approaches may be worthwhile across other medical imaging fields.

## Introduction

Medical imaging for precision medicine relies on biomarkers that capture patient and disease characteristics accurately, efficiently, reproducibly and interpretably. By tradition, the process to establish a biomarker starts with hypothesis generation based on professional experience or theoretical motivation, and concludes with hypothesis testing in specifically designed experiments, for instance by demonstrating the linkage between a marker and clinical outcomes. However, human experts are limited in discovering novel biomarkers because current dogmas may hinder unbiased hypothesis generation, or simply because they may not comprehend the phenotypes of patients and diseases in their full complexity.

Recently, artificial intelligence (AI) has made a powerful entry into medical imaging by automatically replicating specific human tasks of biomarker identification and quantification with superhuman accuracy. For instance, artificial neural networks could autonomously diagnose skin cancer^1^, triage referable retinal diseases^2^ and provide automated diagnoses of chest x-rays^3^ or retinal images^4^. When deep learning was targeted to clinical endpoints, it even enabled prediction of systemic cardiovascular parameters from photographs of the back of the eye^5^. However, these so-called supervised deep learning approaches have critical disadvantages because they can only find what is defined *a priori* by human experts, thus being limited to known biomarkers, and they scale poorly due to the need for labor- or cost-intensive ground-truth training data in the range of several tens of thousands of known samples.

To surpass these limitations, in this paper we explore a paradigm-shifting concept of mining complex high-dimensional medical imaging data. Instead of manual labeling and supervised learning, we propose an unsupervised deep learning architecture particularly designed for OCT representations for unbiased biomarker discovery^6^. We introduce an AI algorithm that teaches itself to capture the most characteristic local structural markers in retinal images, which represent the main patterns of light-tissue interaction as these images are acquired. In a second step, we achieve an extremely compact description of the impractically complex three-dimensional retinal scans. To validate our method, we demonstrate that the obtained biomarker candidates correlate better with clinical outcomes than conventional retinal features.

## Methods

### Retinal imaging by optical coherence tomography

In the field of ophthalmology, modern retinal imaging is performed by optical coherence tomography (OCT), an affordable, non-invasive imaging technique that acquires high-resolution three-dimensional images within instants^7^. It has become the most important diagnostic test in ophthalmology, with approximately 30 million procedures annually or an OCT scan taken every few seconds worldwide^8^. A major issue is that OCT volumes (similar to computed tomography or magnetic resonance imaging scans) contain several hundreds of sections, showing a wide range of tiny retinal changes. These giant datasets have to be interpreted manually by ophthalmologists as part of their daily practice. Moreover, as a relatively new technology, the specific relevance of many of these potential biomarkers remains unclear. This leaves researchers and practitioners overwhelmed by millions of images and a lacking consensus regarding the relevant imaging biomarkers for an efficient management of the leading eye diseases of modern times such as diabetic retinopathy and age-related macular degeneration. This provides a suitable setting for the development and validation of the presented biomarker discovery method.

### Deep learning pipeline

**Figure 1:**
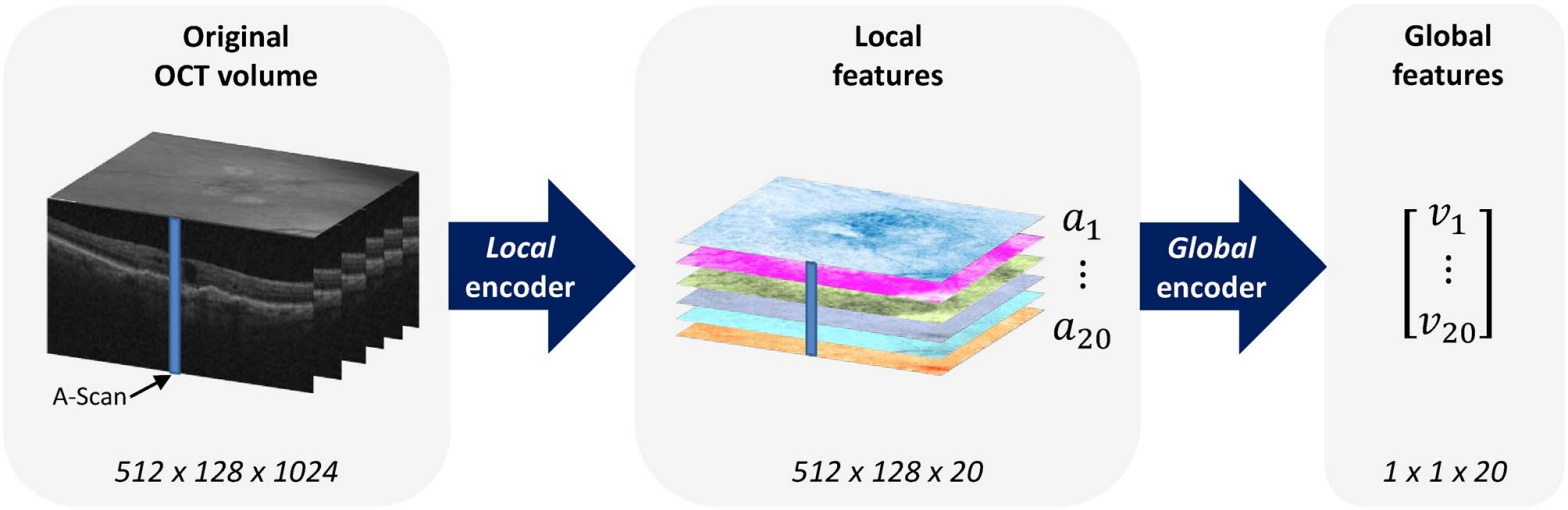
Flow-chart of the proposed two-level deep learning pipeline. In each step, an auto-encoder learns to encode the input data in a lower-dimensional embedding. First, the local encoder transforms each A-Scan into a 20-dimensional local representation, resulting in 20 2D feature maps. This local representation forms the input of the second stage, the global encoder. The global features provide a compact representation of an entire three-dimensional dataset in only 20 numbers.

The proposed AI pipeline (Fig. 1) consists of two auto-encoders to capture (a) the most important local features in the 3D image stack; and (b) a compact global description of the features obtained in the previous step.

In principle, an auto-encoder comprises two sequential deep neural networks. The first (encoding) network is trained to produce high-level low-dimensional descriptors of input data (e.g., an image), while the second (decoding) network is trained to reconstruct the original input data from the high-level description provided by the encoding network. If the reconstruction is accurate (i.e. if the output of the decoder matches the input of the encoder), we can assume that a meaningful high-level representation (or embedding) of the input data has been learned. These learned features serve as novel biomarker candidates in our experiments.

**Figure 2:**
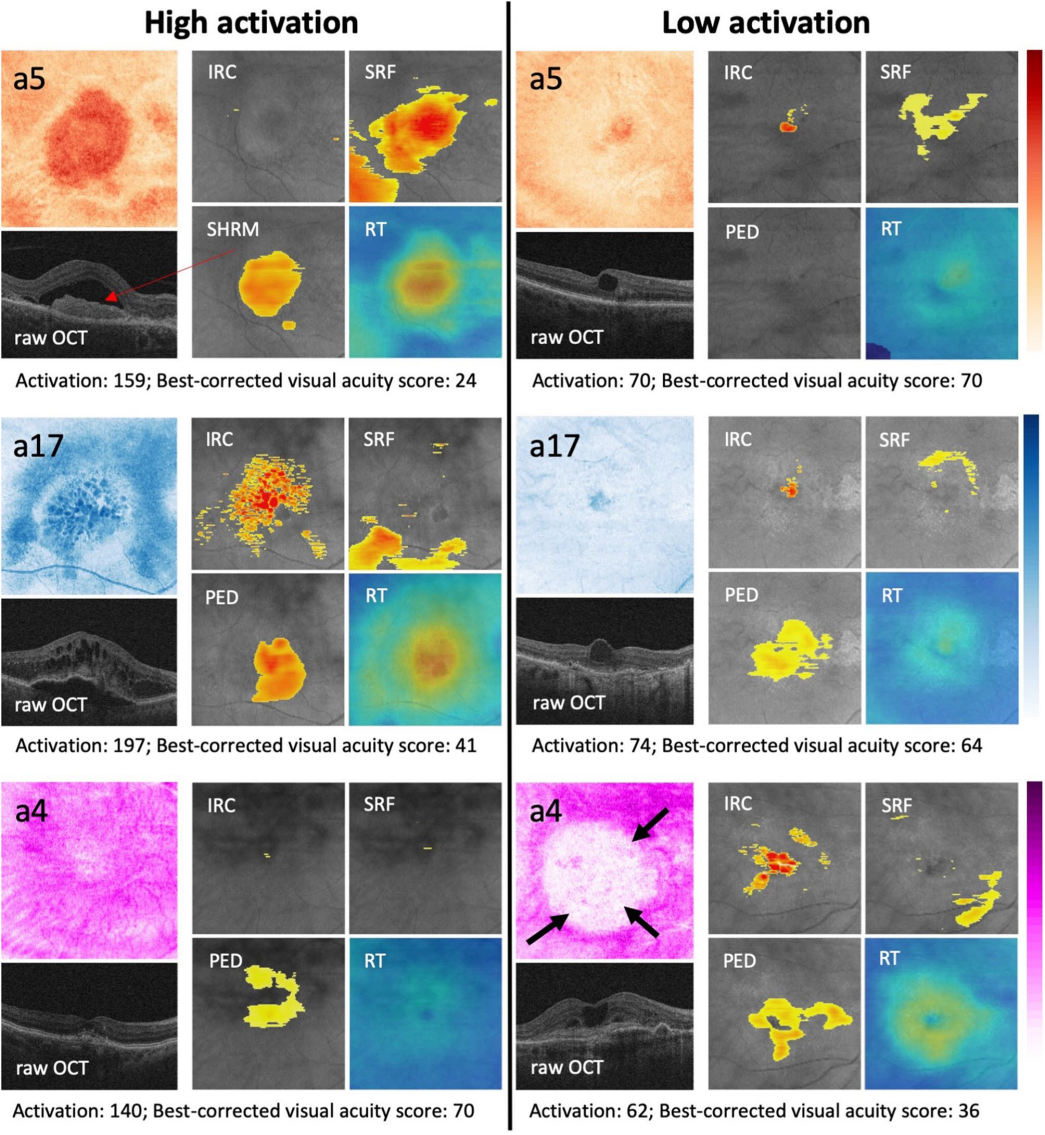
Representative examples of feature maps obtained by the local embedding. The composites to the right of each column show heatmaps of conventional biomarkers obtained by validated automated image segmentation algorithms^11, 12^. High and low activation of the detected new biomarkers with concomitant visual function are shown side-by-side. *Top row:* Feature (**a5**) demonstrates a pronounced negative structure-function correlation, despite a low correspondence to retinal fluid, which is the conventional marker attributed a high relevance for vision. We assume that this biomarker candidate corresponds to subretinal hyperreflective material (arrow). *Middle row:* Feature (**a17**) demonstrates the best correlation with markers of exudation as conventionally measured in OCT. An excellent correspondence is for instance observed for intraretinal cystoid fluid (compare the lobulated pattern). *Bottom row:* Feature (**a4**) represents a new subclinical biomarker candidate discovered in this work (arrows). The marker does not intrinsically correspond to previously reported clinical entities in OCT images. Remarkably, a positive correlation between the activation of a4 and visual function markers was noted. Color bars indicate the activation level from maxiumum (dark) to minimum (light). IRC, intraretinal cystoid fluid; PED, pigment epithelial detachment; RT, retinal thickness; SRF, subretinal fluid; SHRM, subretinal hyperreflective material.

OCT images are acquired by scanning a laser beam tomographically across the retina and sampling the light-tissue interaction at each individual scanning location. Thus, we applied the first auto-encoder on these individual scanning locations resembling vertical signal columns (A-scans, $1\times 1\times 1024$) to learn a 20-dimensional embedding of the local light-tissue interaction. Thus, in this step we receive 20 descriptors of local retinal morphology at each A-scan location. The activation of the 20 learned local features can be displayed and interpreted as feature maps (Figs. 1 and 2). Using these features, we then applied the second auto-encoder on 3D volumes comprised of the obtained local embeddings ($512\times 128\times 20$) and learned a 20-dimensional embedding of the full volumes (Fig. 1). Thus, for each OCT scan we finally receive 20 global features that represent the main spectrum of morphologic patterns of the 3D image data set.

### Clinical datasets for algorithm training and biomarker validation

The auto-encoders were trained on a clinical data set consisting of 54,900 OCT volume scans ($512\times 128\times 1024$ voxels) of 1094 patients enrolled in a classic randomized clinical trial described elsewhere and registered at clinicaltrials.gov with the identifyer NCT00891735^9^. To validate the received biomarker candidates, we used the baseline condition, when all patients presented with treatment naive neovascular age-related macular degeneration in the study eye, and evaluated the correspondence of the identified biomarker candidates with clinically established markers. These included markers of visual function (best corrected visual acuity and low luminance visual acuity), markers of retinal morphology as conventionally measured from OCT (retinal thickness, volume of intraretinal and subretinal fluid, volume of pigment epithelial detachment) as well as measures of disease activity obtained by fluorescein angiography, a conventional, invasive dye-based investigation (total area of lesion, total area of leakage)^10^. We also evaluated qualitatively whether any of the learned, new markers corresponded with established clinical markers as reported previously^10^.

As an additional external test set, we randomly selected 100 baseline OCT volume scans ($512\times 128\times 1024$ voxels, Zeiss Cirrus) of 100 patients with neovascular AMD from our image database, and performed regression analysis as described in Section “Statistical analysis”.

All study procedures were conducted in accordance with the tenets set forth in the Declaration of Helsinki and following Good Clinical Practice guidelines. All patients provided written informed consent before enrollment into the clinical trial. For the retrospective analysis of the image data, approval was obtained by the Ethics Committee at the Medical University of Vienna, Austria.

### Statistical analysis

#### Evaluation using correlation

**Figure 3:**
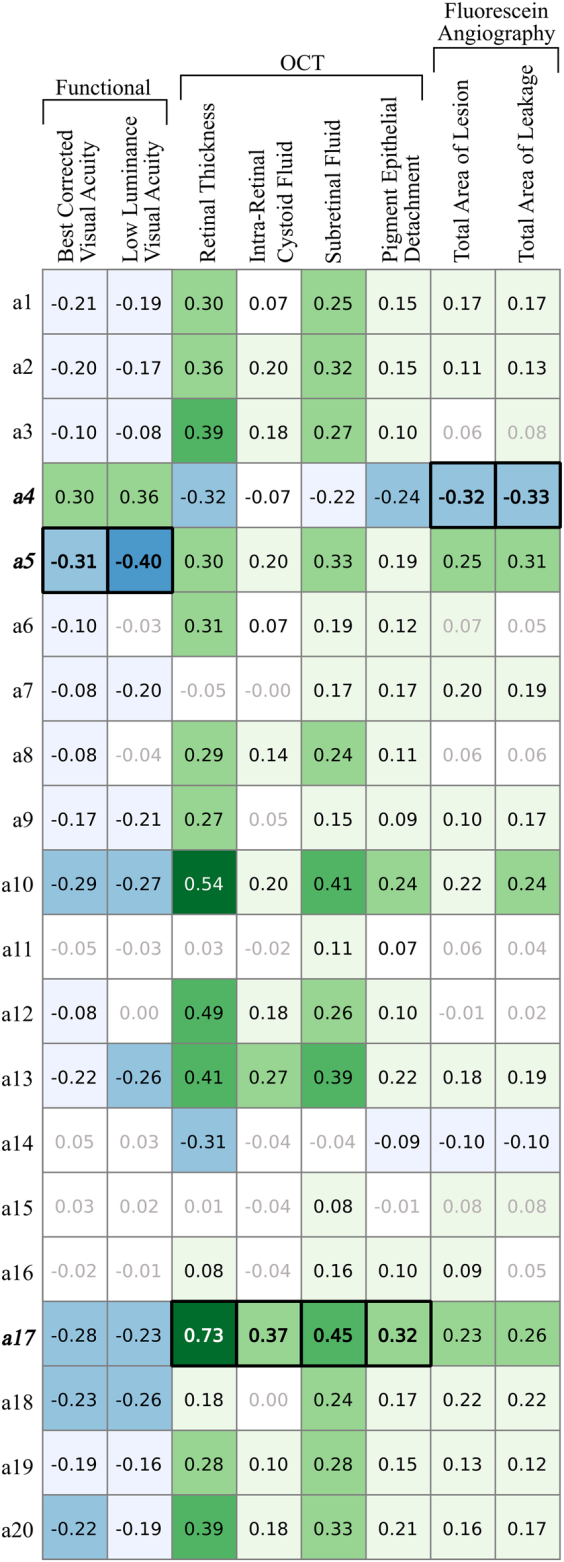
Univariate Pearson correlation coefficients between the 20 identified unsupervised local features (**a1–a20**) and functional variables as well as measures of disease activity by OCT and fluorescein angiography. Green colour indicates a positive, and blue colour a negative correlation. The level of correlation is colour coded, and the strongest correlation for each variable are shown in boxes. Correlations with no significant difference from 0 are greyed out.

For validation of the newly learned features, two different representations were selected as input for our quantitative evaluation: First, to enable a comparison with the same number of features, for each local A-Scan feature, the mean was calculated across the volume and used as feature representation of the entire OCT scan (20 dimensions). Secondly, the global feature vector (20 dimensions) was used. We computed the Pearson correlation coefficient of the detected features with the conventional biomarkers and clinical outcomes described above. The correlation coefficients were computed utilizing the available information of all 1,094 patients. Additionally, we conducted hypothesis tests to evaluate if the correlation coefficients were significantly different from zero. Since this was an explorative study, we did not perform correction for multiplicity testing in order not to increase the type II error (missing an effect that is present). Results are presented in Fig. 3 and Supplementary Table S1, where correlations with no significant difference from 0 are shown greyed out.

#### Evaluation using machine learning regression

For the evaluation using regression models, the treatment naive baseline study eye OCTs described above where randomly divided into training and test sets of 985 and 109 patients, respectively. For each of the above-mentioned known markers, we trained a multiple linear regression model, using the learned 20-dimensional features as input. Elastic net regularization with 5-fold cross-validation was used to determine the optimal hyper-parameters ($\alpha =[0.001 0.01 0.1 0.3 0.5 0.7 0.9 1]$). The performance of the final model was evaluated on the test set.

For comparison against the new markers, conventional OCT markers obtained by automated image segmentation methods, as described in Section “Descriptive power of novel unsupervised features versus conventional features” (21 dimensions) were used as variables to predict visual function (best corrected visual acuity, low luminance visual acuity), using the same settings for the linear regression model as described above. We only predicted the visual function variables for the comparison model, since the conventional morphological markers were used as input here. To test if the performance of the regression model (using global features) was statistically significantly better than the comparison model (using conventional features), we performed a two -sided Wilcoxon signed-rank test with respect to differences in absolute errors, at a significance level of $\alpha =0.05$.

As an additional validation, we applied the obtained linear regression model for prediction of best corrected visual acuity on the external test set.

### Role of the funding source

The funding organizations had no role in the study design, in the collection, analysis, and interpretation of data; in the writing of the report; and in the decision to submit the paper for publication.

## Results

### Local features

The 20 learned unsupervised local features (a1–a20) captured the local morphologic patterns in the OCT data to a high degree and corresponded well to conventional OCT features, but also provided previously unknown features, i.e. subclinical biomarker candidates that had not been considered yet in clinical practice (Fig. 2). The most relevant features are analyzed in detail below. As an independent validation of the new markers, univariate correlations between the average activation of the individual features per OCT volume and the validation metadata are presented in Fig. 3. In general, correlations were stronger for anatomical metadata (r up to 0.73) than for functional metadata (r up to $-0.40$).

Machine learning regression was performed to evaluate the capability of all combined local features to represent retinal morphology, visual function and disease activity. Results of the prediction for functional and anatomical metadata is shown in Table 1.

**Table 1:** Machine learning prediction of functional and morphological target variables from local and global features.

	Visual function		Optical coherence tomography				Fluorescein angiography	
	BCVA (letter score)	LLVA (letter score)	RT ($\mu \hbox {m}$)	IRC (nl)	SRF (nl)	PED (nl)	Lesion area ($\hbox {mm}^{2}$)	Leakage area ($\hbox {mm}^{2}$)
**Local features**								
$R^{2}$	0.26	0.44	0.65	0.09	0.44	0.20	0.27	0.22
MAE	$9.3\pm 7.1$	$10.3\pm 6.5$	$10.6\pm 11.0$	$62\pm 48$	$333\pm 3.3\hbox {e}6$	$300\pm 248$	$1.2\pm 1.0$	$1.3\pm 1.0$
**Global features**								
$R^{2}$	0.29	0.46	0.64	0.19	0.27	0.28	0.21	0.15
MAE	$8.9\pm 7.3$	$9.7\pm 7.0$	$10.9\pm 11.0$	$54\pm 50$	$342\pm 412$	$286\pm 237$	$1.4\pm 1.0$	$1.3\pm 0.8$

For each outcome variable, the coefficient of determination (R$^2$) and mean absolute error (MAE) are shown. BCVA, best-corrected visual acuity; IRC, intraretinal cystoid fluid; LLVA, low luminance visual acuity; nl, nanoliter; PED, pigment epithelial detachment; RT, retinal thickness; SRF, subretinal fluid.

### Interpretation of selected local features

The features with the largest correlation coefficients for each individual meta-variable are further analyzed below and presented in detail in Fig. 2. Feature **a5** achieved the best correlation with functional target variables, i.e. best corrected visual acuity ($\hbox {r} = -0.31$) and low luminance visual acuity ($\hbox {r} = -0.40$). Interestingly, **a5** did not show strong correlations with the quantified morphological variables such as retinal thickness or fluid. However, **a5** visually corresponded to hyperreflective subretinal lesions that may represent subretinal fibrosis with photoreceptor function loss (Fig. 2).

Feature **a17** corresponded best to the conventional fluid-related markers in OCT images, including retinal thickness ($\hbox {r} = 0.73$), intraretinal cystoid fluid volume ($\hbox {r} = 0.37$), subretinal fluid volume ($\hbox {r} = 0.45$) and pigment epithelial detachment volume ($\hbox {r} = 0.32$). Figure 2 illustrates the excellent topographic correspondence between **a17** and segmentations of intraretinal cystoid fluid and subretinal fluid. The retinal vasculature was also captured by **a17**.

Feature **a4** demonstrated the highest (negative) correlation with conventional features obtained by fluorescein angiography. It also surprisingly revealed a markedly positive correlation with retinal function, i.e. $\hbox {r} = 0.30$ with best-corrected visual acuity and $\hbox {r} = 0.36$ with low luminance visual acuity. This marker was negatively correlated with the known OCT markers. Clinically, the feature could not be linked by retina specialists to any of the markers reported in current literature^10^. Thus, our deep learning network identified a hitherto unconsidered subclinical biomarker candidate with a high relevance for visual function. The marker captured the typical pattern of the large choroidal vasculature, and in patients with lower visual acuity showed central punched out regions of low activation (Fig. 2, black arrows).

### Global features

The second autoencoder provided 20 global features per OCT volume scan (**v1–v20**). The univariate correlation of the features with functional and morphologic metadata is shown in supplementary Table S1. The global features do not contain interpretable spatial information; thus, a correlation to image structures similar to the local features is not possible. Generally, the univariate correlations between the global features and clinical metadata were slightly less strong compared to the local features.

Again, multivariate regression analysis was carried out to investigate the capability of all global markers combined to represent retinal morphology, visual function and disease activity. Results of the regression analysis are provided in Table 1. In general, the global features captured the variability in the metadata similarly well as the local features; however, while using a much simpler description of the OCT data.

When the multivariate regression analysis was carried out on the external validation set, the global markers correlated with best corrected visual acuity with an $\hbox {R}^{2} = 0.21$ and a mean absolute error of $10.9\pm 9.2$ letters.

### Descriptive power of novel unsupervised features versus conventional features

We further evaluated the descriptive power of features obtained by the newly developed image analysis approach against conventional biomarkers. For this experiment, we compared the prediction model for visual function based on the new features (Table 1) against a separate prediction model based on traditional markers, i.e. the following variables: Intraretinal cystoid fluid (volume and area), subretinal fluid (volume and area), pigment epithelial detachment (volume and area) and mean retinal thickness. Each of these features was quantified using previously published segmentation approaches^11, 12^ in the central 1mm cylinder centered on the fovea centralis, the 1-3mm ring and the area outside the 3mm ring, resulting in 21 variables for a fair comparison to the 20 unsupervised variables. It is important to note that only the used input features were different (20 learned features vs. 21 conventional markers), while the same linear regression model framework was used for prediction. Using the conventional variables, the quantitative results were $\hbox {R}^{2} = 0.20$ (MAE: $9.3 \pm 7.3$) for best corrected visual acuity ($\hbox {p} = 0.04$ against novel global features with MAE $8.9 \pm 7.3$), and $\hbox {R}^{2} = 0.29$ (MAE: $11.5 \pm 7.9$) for low luminance visual acuity ($\hbox {p} = 0.1$ against novel global features with MAE $9.7 \pm 7.0$). This means that the model based on the new global features achieved a statistically significantly better performance in predicting best corrected visual acuity than the comparison model using conventional features.

## Discussion

Supervised deep learning based on manually labelled input data can successfully replicate the behavior of human experts in relatively simple, but labor-intensive tasks such as in diagnosing skin cancer^1^, triaging retinal OCT scans^2^ or outlining particular lesions on medical images^11^. However, it has critical limitations, including (1) bias introduced by the underlying domain knowledge used to generate the man-made training data, and (2) limited scalability due to often prohibitively large amounts of annotated data required. This conventional AI approach also does not allow to discover hidden, subclinical biomarkers in the data. Some of the limits of supervised deep learning have been elegantly circumvented by reinforcement learning, where, for instance, the computer program AlphaGo Zero achieved superhuman performance in playing the game Go by solely being taught the game rules^13^. In medical imaging however, diagnostic procedures and decisions are not nearly as clear as the rules of a board game, and novel approaches are required particularly as therapeutic implications are often controversial and real world outcomes (particularly in the field of retina) are generally poor. By introducing unsupervised deep learning to medical image analysis, we create a rigorously data-driven analytical tool that is (1) unbiased because it does not rely on human-defined features or hypotheses, and (2) scalable at will because it does not require annotated training data. Our unsupervised deep learning pipeline identified subclinical biomarker candidates in a large-scale dataset that were as good as, or better, in representing the visual acuity of patients than conventional manually defined features measured by state-of-the-art image segmentation methods. We believe our method produces biomarkers that are characteristic of the data, unbiased, compact, task-independent, and easy to obtain.

One major advantage of unsupervised learning is that it automatically detects the most characteristic image features in a dataset, while remaining invariant to any prefabricated and hence biased medical hypotheses. In our experiments, the deep learning algorithm captured the main local biomarkers conventionally used in OCT interpretation, including retinal thickness, intraretinal cystoid fluid, subretinal fluid and pigment epithelial detachment^10^. In addition, it recognized subretinal hyperreflective lesions (marker a5), which are thought to represent incipient fibrosis, as an important feature unrelated to exudation^14^. In fact, this particular feature showed the strongest correlation with visual function in our cohort; albeit currently not being considered as an endpoint in trials for retinal therapeutics. Thus, our disruptive approach of an unbiased biomarker search may be useful in identifying, defining and prioritizing targets and endpoints for the development of new compounds and interventions. In addition to representing the main known characteristics, our method may also be used to discover new marker candidates in image data. To complement the “usual suspects”, the deep learning algorithm identified a new feature (a4), which demonstrated a pronounced positive correlation with visual acuity in our cohort of patients. Further research may be directed at identifying anatomical correlates for this subclinical marker, such as intact neurosensory structures which do not attract any attention in current clinical trials despite morphological appearance. Currently, we are unable to pinpoint individual local markers to a particular anterior-posterior location in OCT A-scans, and therefore the exact origin of the feature activation is yet unknown. Nevertheless, biomarker discovery such as reported here may become an important aspect in medical image interpretation as conventional markers are regarded to have substantial weaknesses in reflecting patient-centered outcomes (such as visual acuity), and pivotal drug developments fail presumably also due to the lack of reliable endpoints^15, 16^.

Obviously, not all local markers represent clinically relevant information—similar to the OCT image itself. For instance, feature a11 showed very low correlations with the provided meta-information, while showing homogenous activation patterns across images. We may speculate that some of our features, including a11, capture image characteristics such as noise, that are part of the image, but do not relate to individual biomarkers. Future work may address the automated establishment of the number of dimensions in the auto-encoder embedding and thus analyze how many individual features are required to represent an image dataset comprehensively.

The second step of the unsupervised embedding resulted in a characteristic, compact representation of complex three-dimensional medical imaging datasets. Without human-made limitation to particular variables or measurements, our algorithm provides 20 quantified global features for each volume that represent the major morphologic patterns in the image data, as opposed to an unmanageable amount of 67m ($512\times 128\times 1024$) voxels in the native image. Despite this heavy compression, the resulting measurements still correlate well with visual acuity, conventional markers on OCT, and multimodal markers of disease activity (e.g. on fluorescein angiography), and surpass conventional OCT markers obtained by automated image segmentation methods in representing visual function, as shown by the results in Section “Descriptive power of novel unsupervised features versus conventional features”. Once validated in prospective studies, we believe that unbiased, manageable descriptions of OCT such as the one presented here may be applied in clinical and research practice because they could significantly facilitate the interpretation of complex imaging data, and make therapeutic decision making based on imaging studies at the same time simpler, as well as more reliable.

In our experiments, we achieved a coefficient of determination of $\hbox {R}^{2} = 0.29$ and $\hbox {R}^{2} = 0.46$ between unsupervised global features and best-corrected and low-luminance visual acuity, respectively, in a large cohort of patients with neovascular age-related macular degeneration at the native stage. Although these correlations may appear moderate, these results actually outperform those previously reported in the literature for large datasets, and were indeed superior to the correlations achieved by using conventional OCT markers such as fluid volume, which highlights the value of our approach^17^. When applied on an external test set, prediction accuracy was reduced to $\hbox {R}^{2} = 0.21$, which may be explained by different patient populations with differing distributions of best-corrected visual acuity values in the two datasets.

Interestingly, the correlation with low-luminance visual acuity was consistently larger than with best-corrected visual acuity. Previous studies have shown impairment in low-luminance visual acuity in patients with age-related macular degeneration that exceeds the deficits seen in best-corrected visual acuity^18-20^. Possibly, morphologic changes on OCT correspond better to low-luminance visual acuity because it is a more sensitive measurement of visual dysfunction in the macular area.

Unsupervised deep learning has previously been leveraged in medical imaging. For instance, an unsupervised learned representation of local 2D image patches was used for the task of mammography risk scoring^21^. In the context of ophthalmic imaging, researchers have proposed algorithms to identify abnormal tissue patterns by learning the characteristic appearance of normal tissue^22, 23^. In such an approach, anomalous regions can be analyzed further to establish clusters of biomarkers allowing to define marker categories. In contrast, we propose to learn both local and global high-level descriptions of images, which are not restricted to anomalous structures, to provide a compact representation of entire volumes, and omit the need to prepare a dataset of normal patients. We believe that this makes our approach a valuable tool for hypothesis generation and biomarker identification, supporting a critical shift of mindset in medical image analysis. Namely, it expands the conventional biomarker evaluation strategy from supervised automation of expert annotation in known anomalies to an unrestricted unsupervised exploration of large-scale datasets.

Whenever image data are compressed to high level representations, topographic information of the represented biomarkers is reduced. In our global embedding, visualization of the features is not possible any longer because they do not contain any spatial information. Thus, it is challenging to interpret the individual biomarkers and their contributions to the variability of retinal morphology. These difficulties in interpreting the mechanisms of the deep learning model constitute the main limitation of our proposed algorithm. From a clinical perspective, it has always been desirable to clinically understand the steps taken by a model to reach a particular decision^24^. However, if doctors wish to augment their practice by artificial intelligence, these traditional paradigms may need to be revisited particularly as the hardware technology of image acquisition has long surpassed the feasibility of expert-based definition of features and assessment of imaging studies.

In this paper we introduced unsupervised deep learning to analyze high-resolution, three-dimensional retinal images without human-introduced bias. We presented novel auto-encoder based technology to capture the most relevant local structural biomarkers, including discovery of a new subclincial marker candidate. In a second embedding, we obtained a compact global description of the complex three-dimensional retinal scans, which nevertheless correlated better to visual acuity of patients than established artificial-intelligence based measurements. Once validated in additional, independent datasets, unsupervised machine learning and the resulting biomarkers may be employed in medical image analysis in retinal imaging and beyond.

## Supplementary Information

Supplementary Information.

## Data Availability

The HARBOR study data are property of Genentech and will be made available upon reasonable request.
