# Supplementary material for: Unbiased identification of novel subclinical imaging biomarkers using unsupervised deep learning
Source: Sci Rep. 2020 Jul 31;10:12954. doi: 10.1038/s41598-020-69814-1 (PMC7395081; doi:10.1038/s41598-020-69814-1)
Supplement: Supplementary file 1 — Supplementary Information. [file 41598_2020_69814_MOESM1_ESM.pdf]

Supplementary Information for

Unbiased identification of novel subclinical imaging biomarkers using unsupervised deep learning

Sebastian M. Waldstein[+], Philipp Seeböck[+], René Donner, Amir Sadeghipour, Hrvoje Bogunović, Aaron Osborne, Ursula Schmidt-Erfurth*[1]

[+]these authors contributed equally to this work

*corresponding author. *email:* ursula.schmidt-erfurth@meduniwien.ac.at

This PDF file includes:

Supplementary text
Figures S1 to S2
Table S1
SI References

Supporting Information Text

1. Supplementary Table

	Functional		OCT				Fluorescein Angiography	
	Best Corrected Visual Acuity	Low Luminance Visual Acuity	Retinal Thickness	Intra-Retinal Cystoid Fluid	Subretinal Fluid	Pigment Epithelial Detachment	Total Area of Lesion	Total Area of Leakage
v1	-0.13	-0.13	0.13	0.07	0.14	0.04	0.02	0.01
v2	-0.32	-0.27	0.52	0.40	0.26	0.10	0.18	0.23
v3	-0.05	-0.03	0.11	0.25	-0.00	-0.03	0.01	0.01
v4	-0.07	-0.04	0.23	0.12	0.19	0.18	0.13	0.11
v5	-0.15	-0.07	0.44	0.21	0.26	0.16	0.11	0.10
v6	**-0.33**	**-0.30**	**0.67**	0.38	0.39	**0.31**	**0.27**	**0.31**
v7	-0.09	-0.08	0.24	0.13	0.20	0.05	0.04	0.04
v8	-0.24	-0.23	0.56	0.27	**0.43**	**0.31**	0.19	0.19
v9	-0.09	0.05	0.33	0.32	0.10	-0.03	-0.08	-0.07
v10	-0.30	-0.23	0.41	**0.42**	0.19	0.09	0.10	0.13
v11	-0.20	-0.17	0.42	0.15	0.29	0.16	0.16	0.17
v12	-0.07	-0.04	-0.02	0.08	-0.00	-0.00	0.06	0.05
v13	-0.20	-0.13	0.23	0.13	0.16	0.09	0.13	0.15
v14	-0.28	-0.24	0.41	0.31	0.33	0.17	0.16	0.19
v15	0.03	0.05	0.01	-0.04	0.04	-0.00	0.00	-0.02
v16	-0.21	-0.18	0.28	0.41	0.07	0.11	0.06	0.10
v17	-0.23	-0.14	0.37	0.28	0.11	0.09	0.05	0.09
v18	-0.09	-0.03	0.16	0.11	0.09	-0.05	0.04	0.03
v19	-0.25	-0.20	0.41	0.31	0.24	0.15	0.23	0.23
v20	-0.29	-0.26	0.46	0.41	0.29	0.20	0.18	0.21

Table S1. Supplementary Table S1: Univariate Pearson correlation coefficients between the global features (v1 – v20) and functional variables as well as measures of disease activity by OCT and fluorescein angiography. Green colour indicates a positive, and blue colour a negative correlation. Correlations with no significant difference from 0 are shown greyed out.

2. Supplementary Methods

A. Background and approach. In optical coherence tomography (OCT) an interferogram is obtained at a specific point of a sample, yielding an A-Scan containing one-dimensional information (along the z-axis) (1). The A-scan data thus represent the condition of the retina at that specific position in the eye. By scanning the measurement beam across the sampling area, millions of A-scans are concatenated to form entire volume scans. It is this multi-step data acquisition which motivates the reasoning behind our proposed approach. Instead of trying to find an embedding for a volume in a single step, we construct two separate embeddings as depicted in Figure 1 of the main manuscript that reflect the underlying process of OCT acquisition as well as the basic anatomy of the retina. In the first level, we learn a compact embedding of A-Scans and therefore of the local condition of the retina, using a fully connected auto-encoder. In the second level, a convolutional auto-encoder is used to learn a global representation of whole OCT volumes based on the embedding obtained in the first level, resulting in a massive reduction of dimensionality.

B. Dataset. The experiments reported in this paper were conducted on a dataset consisting of 54,900 OCT volume scans of 1,094 patients enrolled in a randomized clinical trial (2). The volumes were acquired using Cirrus OCT devices (Carl Zeiss Meditec, Dublin, CA, USA) and had a voxel dimensionality of 512 × 128 × 1024, covering a physical area of 6mm × 6mm × 2mm, with a voxel spacing of 11.7 micrometer × 46.9 micrometer × 2 micrometer. The dataset was randomly divided into a train (90%) and test set (10%) with 985 and 109 patients, respectively. There was no overlap of patients between those two sets.

C. Data preprocessing. To reduce the large amount of speckle noise inherently present in OCT data, we use Bilateral Grids due to their fast runtime and easy implementation, on the individual B-Scans. We perform a single pass of filtering to reduce noise while retaining subtle details (3). The position of the retina along the A-Scan is not fixed and depends on patient position during acquisition. To be invariant to this translation we compute a one-dimensional Fast Fourier Transform (FFT) of the A-Scan and discard the phase information by keeping only the magnitude of the complex FFT signal. Due to the resulting symmetry of the real-valued signal we only keep a vector of length 512 of the FFT amplitudes per 1024-long A-Scan.

D. Deep unsupervised learning of local features. Auto-encoders are trained without any labels and consist of two parts, the encoder and the decoder. During training, the input is encoded by the encoder into a low-dimensional embedding, and subsequently decoded by the decoder to reconstruct the original input. The underlying assumption is that the auto-encoder has to learn a meaningful compact high-level representation of the data to be able to perform accurate reconstruction. In Figure S1 and Figure S2, information within each auto-encoder always flows from the left to the right, with the embedding being the lowest-dimensional state in the middle of the stack. In the first stage of our framework (Figure S1), the A-Scan auto-encoder AE_1 is composed of three simple fully connected layers ([256/64/20] channels), with a weight matrix W_l, a bias vector b_l and an activation function σ:

$$y_l = \sigma(W_l x_l + b_l). \quad [1]$$

The sizes of the layer on both sides of the embedding are mirrored, and the weight matrices of two corresponding layers are tied: $W_l = W_l^T$. Throughout this work the activation function σ is set to be the exponential linear unit (ELU) (4), with $\alpha = 1$:

$$f(x) = \begin{cases} x & \text{if } x > 0 \\ \alpha(exp(x) - 1) & \text{if } x \leq 0 \end{cases}, \quad [2]$$

The cost function used to drive the optimization in auto encoders measures the reconstruction error of the final output y given an input vector x:

$$C(x) = \sum (x - y)^T (x - y). \quad [3]$$

Using a randomly sampled subset of all the A-Scans available in the training set (1,600,000 A-Scans), a first auto-encoder is learnt in an end-to-end fashion as proposed in Zhou et al. (ref 28). The A-Scans within each volume are sampled from a Gaussian distribution, implying a higher chance for more centrally-located (clinically relevant) A-Scans to be part of the training subset. After training, only the encoder is used to map the A-Scans of all volumes into the embedding space, yielding the A-Scan features for each A-Scan. The individual A-Scans are processed independently from their position in and membership of any OCT volumes.

E. Deep unsupervised learning of global features. The A-Scan features of each volume are normalized feature-wise (zero mean, unit standard deviation) and concatenated according to their positions in the volume, yielding A-Scan feature volumes, reducing the volume size 50 times from 512 × 128 × 1024 to 512 × 128 × 20. Based on this compressed representation, in the second part of our framework, a deep convolutional auto-encoder is trained from all 49,505 training volumes.

This second auto-encoder AE_2 is composed of one linear down-sampling layer, followed by five convolutional ([64/64/128/256/512] channels) and three fully connected layers ([256/64/20] channels) on the encoder side, and a mirrored structure on the decoder side, as depicted in Figure S2. All layers are followed by the non-linear activation function ELU, and random-region dropout is applied to the input during training (5). Applying the encoder of AE_2 on the A-Scan feature volumes yields a 20-dimensional global feature vector for each volume.

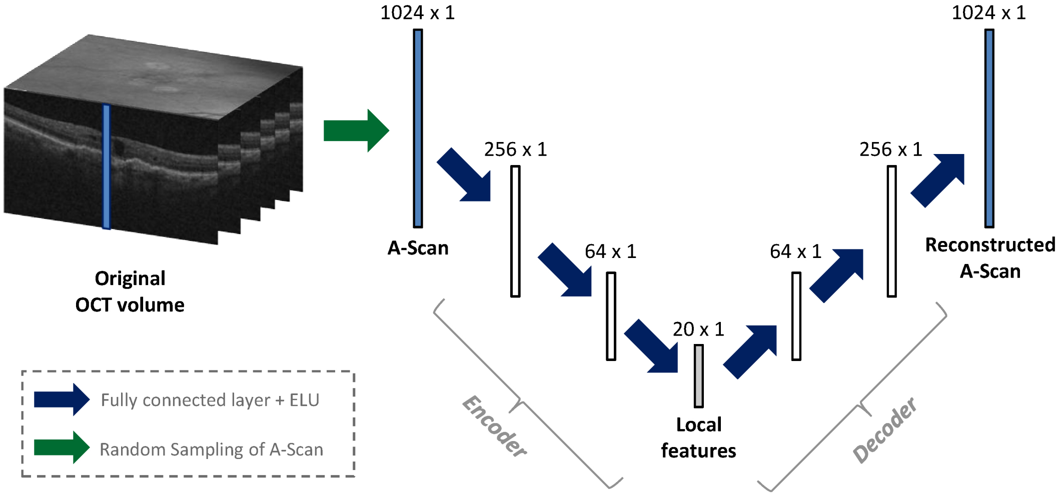

Figure S1. Illustration of the local auto-encoder architecture AE_1. Local features are learned using randomly sampled A-Scans from OCT volumes. During training, A-Scans are reconstructed from the compact representation (20 dimensions).

F. Training details. For the training of the fully connected and the convolutional auto-encoder, we use Adam optimizer with standard parameters. For the former we use a learning rate of 0.0001, early stopping with a maximum of 500 epochs, a minibatch size of 64 and dropout at the input level with a rate of 0.5. For the latter we use a learning rate of 0.0001 for 10 epochs and 0.00001 for 2 epochs, a minibatch size of 8, random-region dropout-factor of 0.25 for the input and ordinary dropout in the first fully-connected layer of AE_2 with a factor of 0.5.

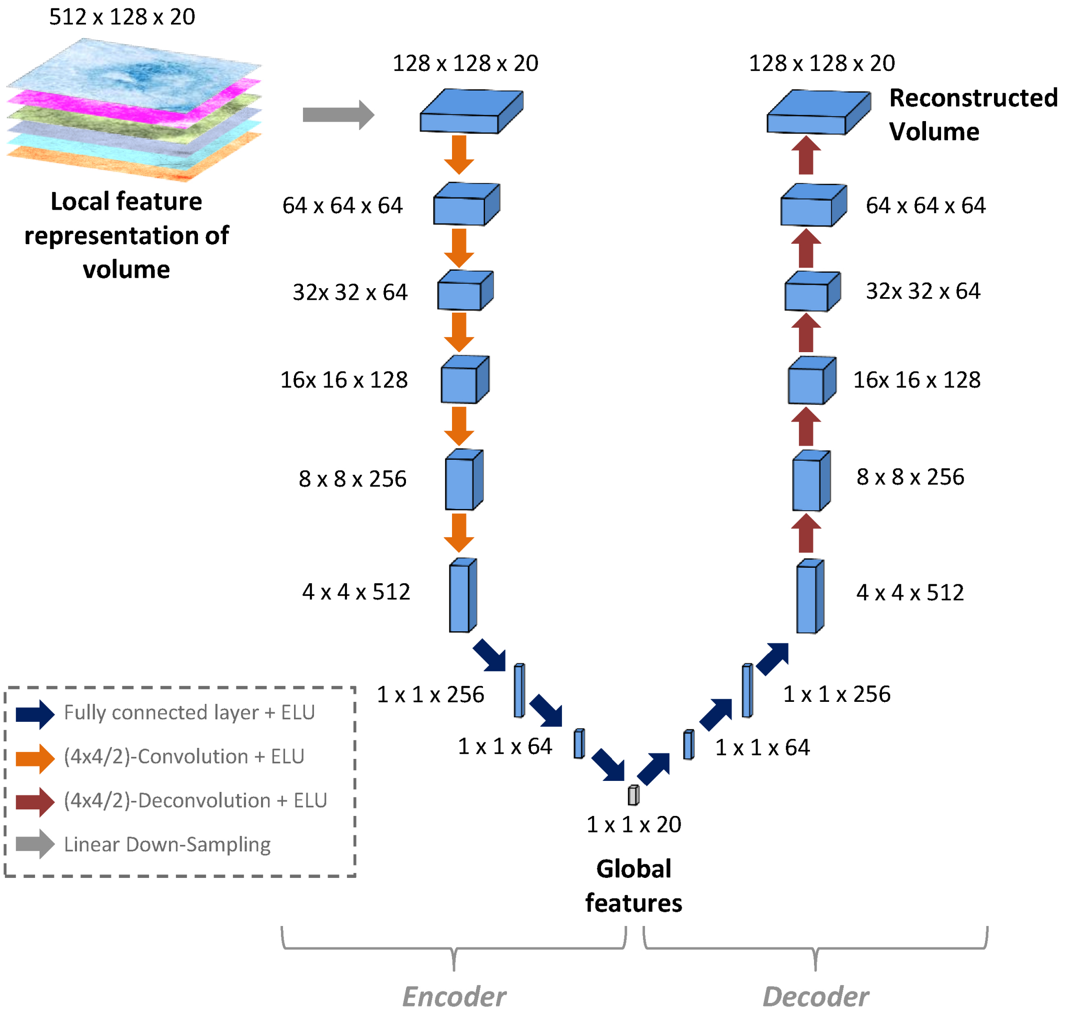

Figure S2. Architecture of the global auto-encoder AE_2. Encoding the local feature representation volume yields a compact global feature embedding, representing the whole OCT volume in only 20 dimensions.

References

1. D Huang, et al., Optical coherence tomography. *Science* **254**, 1178–1181 (1991).
2. BG Busbee, et al., Twelve-month efficacy and safety of 0.5 mg or 2.0 mg ranibizumab in patients with subfoveal neovascular age-related macular degeneration. *Ophthalmology* **120**, 1046–1056 (2013).
3. J Chen, S Paris, F Durand, Real-time edge-aware image processing with the bilateral grid. *ACM Transactions on Graph. (TOG)* **26**, 103 (2007).
4. DA Clevert, T Unterthiner, S Hochreiter, Fast and accurate deep network learning by exponential linear units (elus). *arXiv preprint arXiv:1511.07289* (2015).
5. D Pathak, P Krahenbuhl, J Donahue, T Darrell, AA Efros, Context encoders: Feature learning by inpainting in *Proceedings of the IEEE conference on computer vision and pattern recognition.* pp. 2536–2544 (2016).
